# Conservative Medicine

**Published:** 1862-11

**Authors:** Austin Flint


### CONSERVATIVE MEDICINE.—By Austin Flint, M. D.

From the American Medical Monthly.

"What does the writer mean by *Conservative Medicine?*" This will be the mental inquiry of the reader when the caption of this article meets his eye. It is desirable, first of all, for the writer to explain the subject which he ventures to hope will appear to possess interest enough to lead to a perusal of the pages which are to follow.

The meaning of *Conservative Surgery* is well understood. This phrase has been sufficiently common of late years. The conservative surgeon aims to preserve the integrity of the body. He spares diseased or wounded members whenever there are good grounds for believing that by skillful management they may be saved. He resorts to mutilations only when they are clearly necessary. He weighs carefully the dangers of operations, so as not to incur too much risk of shortening life by resorting to the scalpel. By conservative medicine, I mean an analogous line of conduct in the management of maladies which are not surgical. The conservative physician shrinks from employing potential remedies whenever there are good grounds for believing that disease will pursue a favorable course without active interference. He resorts to therapeutical measures which must be hurtful if not useful, only when they are clearly indicated. He appreciates injurious medication, and hence does not run a risk of shortening life by adding dangers of treatment to those of disease. Such, in brief, is an explanation of the subject of this article. For the phrase, *conservative medicine*, I am indebted to a distinguished friend and colleague, well known as eminently a conservative surgeon.*

During the last quarter of a century a change has taken place in medical sentiment as regards surgical operations. New and grand achievements in surgery seemed formerly to be the leading objects of personal ambition. To borrow a fashionable expression, they were decidedly the rage. Boldness in the use of the knife was the trait in the character of the surgeon which was most highly admired. The history of surgery during the first third of the present century is characterized by the introduction and frequent performance of numerous formidable operations. It was customary to speak of them as brilliant, and the daring surgeon enjoyed somewhat of the *eclat* which belongs to the hero of the battle-field. This analogy was implied when one of the greatest of our American surgeons, wishing to distinguish his most brilliant exploit, styled it his Waterloo operation. The change that has taken place is marked. We hear now comparatively little of terrible operations, and of that sort of heroism which is associated with bloody deeds. What would once have been considered as a degree of courage to be admired, is now stigmatized as rashness. It is an equivocal compliment to say of a practitioner that he is a

*Prof. Hamilton.

bold surgeon. The change, it may be said, is in a measure due to the fact that the great number of new operations which have been introduced since the beginning of the present century leaves but a limited range for further explorations in that direction; but this explanation will go only a little way. The change is one of sentiment. The desire is to preserve the integrity of the body, to avoid mutilations, to incur the dangers of capital operations only when they are imperatively called for—in a word, conservatism has become the ruling principle in surgery. The most important of the most recent improvements in surgery exemplify the influence of this principle on the medical mind.

An analogous change, within the same period, has taken place in medical practice. Formerly, boldness was a distinction coveted by the medical, as well as by the surgical practitioner. "Heroic practice" was a favorite expression, consisting in the employment of powerful remedies, or in pushing them to an enormous extent. The physicians emulated the surgeons in daring. The change is not less marked in medicine than in surgery. We hear now oftener of diseases managed with little or no medication, than of cases illustrating the abuse of remedies. In the treatment of many affections it is not considered necessary to employ measures which, but a few years ago, it would have been considered culpable to withhold. The change, too, is here one of sentiment. We desire to preserve the vital forces, to avoid the perturbations and damaging effects of potential therapeutic agencies—in short, conservatism has become a leading principle in medicine as well as in surgery. The improved method of managing a host of affections will be found to illustrate this fact.

Before proceeding further, let us inquire how the contrast between medical practice at the present moment and a quarter of a century ago, should affect our estimation of medicine. Is medicine disparaged by the changes which have actually taken place? It is not enough to answer this question in the negative. Mutations, when they denote progress, are, of course, desirable. In so far as the contrast shows improvement, medicine at the present moment is deserving of esteem, the more, as the changes are great. It redounds to the glory of medicine that it admits of illimitable progressive changes. In this fact lies the distinctive feature of legitimate medicine as contrasted with illegitimate systems of practice. But, some one may say, is there to be no stability in medicine, no traditional authority, and is reverence for the past to have no influence? If not, where is our ground of confidence in the practice of the present day? And is it not probable that at the end of another quarter of a century mutations will have occurred twice as great as those which have taken place during the last twenty-five years? These questions are to be met fairly and squarely; let us endeavor so to meet them.

Waiving the consideration of what constitutes perfectibility in the *ars medendi*, and whether it be obtainable or not, no one will assert that medicine is now, or ever has been, in a condition not to admit of indefinite improvement. Improvement in its practical applications and results is the great end of the labors devoted, now and hitherto, to the different departments of medical knowledge, viz: anatomy, physiology, animal chemistry, materia medica, pathology, and clinical medicine. We may assume that these labors, thus far, have not been profitless, and, accordingly, that practical medicine has improved. We may assume, also, that there is abundant encouragement to continue these labors, and, hence, that further improvement is to be expected—to what extent it is vain to speculate. It necessarily follows that stability in medicine is not to be counted upon; that the doctrines of to-day have no intrinsic claim to perpetuity; that because they are now in vogue is not a sufficient reason why they should not hereafter be modified or rejected, and that there is, to say the least, no ground to deny the possibility of the changes which are to take place hereafter being a whit less than those which have already taken place.—What then? There are skeptics and scoffers with regard to medicine, and there are many persons who live and thrive by promoting popular distrust of it. It may seem to be giving aid and comfort to the enemies of medicine to concede that its past history abounds in errors, that present errors doubtless abound, leaving ample scope for future improvement. Be it so. We have nothing to do with skeptics, scoffers and charlatans. We are not called upon to repel attacks prompted by ignorance, selfishness and deceit. Yet it is desirable, with regard not only to the interests of the medical profession, but to the welfare of humanity, that medicine should hold its proper place in popular estimation. What, then, is the attitude to be taken as regards the just claims of medicine to public consideration and confidence? A body of men in every generation, from the time of Hippocrates to the present day, in all civilized countries, have conscientiously and industriously labored to acquire knowledge of diseases with reference to the relief of suffering and the prolongation of life. Under a host of difficulties and obstructions, many inherent in the pursuits themselves, and others proceeding from various extrinsic sources, the labors of physicians and their colaborators have continued and still continue. Now, granting that they have advanced slowly and often been led astray, where else can society ever seek for aid in the necessities of illness with a better prospect of success? Granting that they have failed, and still fail, in conferring all the benefit that is to be desired, and that, with the purest intentions, their efforts have been sometimes not only without avail, but hurtful, should the preponderating good be therefore overlooked, and is there any rational alternative but to accept good and submit to the limitations and errors incident to existing knowledge? All that society can claim of medicine in any generation is, the capabilities of the medical science in that generation. All that society can claim of physicians is, that these capabilities shall be understood and judiciously applied. But we are opening up trains of thought which will lead us a long way from our subject, and we must abruptly return to the consideration of conservative medicine.

It is an interesting point of inquiry, whence came the influences leading to conservatism as a principle of medical practice? The answer to this inquiry would not be the same in all countries and sections. It must be admitted that in our country the earliest and fullest development of the principle was in New England. Our New England brethren are fond of dating a new order of medical ideas from the publication of an address, more than twenty years ago, by Jacob Bigelow, on the self-limited character of certain diseases. Not underrating the importance of that publication, the spirit of the oral teachings of James Jackson and John Ware has exerted on the medical mind of New England an influence which can only be appreciated by those who have experienced it. To those who have experimentally the value of their teachings, it is a source of deep regret that the influence of these admirable professors has not been more widely diffused by means of larger contributions to medical literature. British conservatists attribute much to the writings of Dr. Forbes. Among the non-medical observers of the change in practice which has taken place some have been persuaded that it is due to the disciples of Hahnemann, an idea too preposterous to need refutation. The truth is, we are not to look for the causes of the change exclusively in the views emanating from particular persons. It is rather a legitimate result of scientific researches in different directions. If we were to specify circumstances which have more especially been instrumental in leading to the principles of conservatism, we would mention, *first*, the abandonment of the attempt to found a system or theory of medicine after the decline and fall of Brunonianism and Broussaisism; and *second*, the study of diseases after the numerical method with reference to their natural history and laws.

Strange as it appears, the importance of determining by clinical observation the intrinsic tendencies of different diseases as the basis of therapeutics, seems to have been heretofore overlooked. Physicians have acted on the presumption that most diseases do not pursue a favorable course without treatment more or less efficient. This has been, to a still greater extent, the popular belief. The apparent proof of the success of the Hahnemannic treatment rests on this belief. What are the facts already ascertained with respect to the intrinsic tendencies of different diseases? We know that diseases in the management of which, but a few years ago, the physician would not dare to omit potent therapeutical measures, almost invariably end in recovery without any active treatment. Take, as examples, pneumonia limited to a single lobe, and acute pleurisy. It is sufficiently settled that these diseases involve very little danger in themselves, proving fatal only in consequence of complications. The practitioner, therefore, no longer feels obliged to employ blood-letting, mercurialization, cathartics, blisters, etc., in these diseases, with reference to the saving of life. The only question is, do patients pass through these diseases as well without as with such measures of treatment? Clinical observation, following up this inquiry, arrives at results which exemplify conservative medicine.

Our acquaintance with the natural history of the great majority of diseases is, as yet, very incomplete. Knowledge of the tendencies of diseases allowed to pursue their course without active treatment, is not readily acquired. We cannot conscientiously withhold remedies which we have reason to believe may prove useful. Cases are therefore to be slowly accumulated in which, from circumstances not under our control, diseases have been uninfluenced by therapeutic interference. This knowledge, it is evident, is the true point of departure for the study of the effects of remedies as regards the termination and duration of diseases. The information already obtained has rendered the use of powerful therapeutic agencies far less than they were but a few years since. It remains to be seen hereafter what will be the further effect on medical practice of continued researches in this direction.

Conservative medicine assumes that remedial measures, according to their potency, must either do harm or good; that they can never be neither hurtful nor useful. Prior to the advent of conservatism, this important fact was not duly appreciated. Blows were leveled at diseases, but the patient was not enough considered. It did not enter sufficiently into the calculations of practitioners that if successive blows dealt at a disease were misdirected, the effect was not lost, but injury was inflicted in proportion to their force. Hence, it must needs follow that the sick man sometimes encountered, in addition to his malady, assaults not less real because well meant. In this respect, certainly, we have evidence of progress. We are satisfied that we do not err in saying that the most judicious practitioners of the present day accept the following maxims of that eminently conservative physician, Chomel: *first*, that we are not so much to treat diseases, as patients affected with disease; and *second*, that not to do harm, is no less an object of treatment than to do good.

In defining conservative medicine, we have seen that it expresses a characteristic of the improvements in medical practice during the last twenty-five years. Let us now direct our attention to illustrations afforded by some of the different classes of remedial measures. And, first of all, blood-letting suggests itself. How great the change as regards this remedy! Twenty-five years ago it was employed as if it were an innocuous remedy. Practitioners thought much more of the risk of not resorting to it when it was needed, than of the evils of its being needlessly resorted to. Hence, they often acted on the rule inculcated by a medical writer, viz: when in doubt use the lancet. How different the rule of treatment now! Few practitioners of the present day would resort to this remedy in any case in which its appropriateness seemed to them questionable. Why not? Because it has been ascertained to be a spoliative remedy. It causes a disproportionate loss of the corpuscular elements of the blood, which are slowly regenerated. These corpuscular elements are already deficient in many diseases. In short, anæmia and its pathological relations were very imperfectly understood a quarter of a century ago. It is clear now to every one that, if not indicated, blood-letting should never be employed. This simple statement explains, in a great measure, the comparative disuse of blood-letting. The great question now is, whether it is a remedy called for more or less frequently in the management of certain diseases, chiefly the acute inflammations. I do not propose to enter here into a discussion of this question. This much may be said: Clinical observation, which is alone competent to settle the question, has shown that it is a remedy not called for so often or to so great an extent in acute inflammations as was supposed but a few years ago.

[TO BE CONTINUED.]

